# Effects of o,p’-DDE, a Mitotane Metabolite, in an Adrenocortical Carcinoma Cell Line

**DOI:** 10.3390/ph15121486

**Published:** 2022-11-29

**Authors:** Camila Bach, Claudia Rita Corso, Alan de Almeida Veiga, Mariana Martins Paraizo, Lauro Mera de Souza

**Affiliations:** 1 Instituto de Pesquisa Pelé Pequeno Príncipe, Av. Silva Jardim, 1632-Água Verde, Curitiba CEP 80250-060, PR, Brazil; 2 Faculdades Pequeno Príncipe, Av. Iguaçu, 333-Rebouças, Curitiba CEP 80230-020, PR, Brazil

**Keywords:** drug metabolism, cell death mechanism, necrosis, o,p’-DDE, o,p’-DDD

## Abstract

In South Brazil, the incidence of pediatric adrenocortical carcinoma (ACC) is higher than in other regions and countries worldwide. The ACC treatment includes therapy with mitotane, the only adrenolytic drug approved by the FDA. The mitotane metabolism occurs via two main reactions: the β-hydroxylation, which yields the final product o,p’-DDA, and the α-hydroxylation, which will give the final product o,p’-DDE. It is speculated that o,p’-DDE may be an active metabolite since it has a cytotoxic effect on adrenocortical carcinoma cells (H295R). No further studies have been conducted to confirm this hypothesis; however, it was found that mitotane and its metabolites are present at significantly different concentrations in the plasma of the patients. Our study aimed to assess the in vitro effects of o,p’-DDE and o,p’-DDD in cell death pathways, oxidative parameters, and interaction with adrenal CYP’s involved in the steroidogenic process in the H295R cell line. It was found that o,p’-DDE had a different effect than the o,p’-DDD on apoptosis, inhibiting this cell death pathway, but it promotes cell necrosis at higher concentrations. In contrast to o,p’-DDD, the o,p’-DDE did not have effects on the different oxidative parameters evaluated, but exhibited stimulatory interactions with steroidogenic CYP’s, at intermediate concentrations. Therefore, we demonstrated important cell effects of o,p’-DDE; its plasma levels during mitotane therapy should be monitored as an important therapeutic parameter.

## 1. Introduction

Adrenocortical carcinoma (ACC) is an endocrine tumor that affects the adrenal glands, promoting the overgrowth of this organ and increasing the production of steroid hormones, such as androgens and cortisol. In pediatric patients, this leads to early virilization (acne, pubic hair, hirsutism, increased muscle mass) and Cushing’s syndrome. The overall incidence of ACC in adults is 1.7 to 2 cases per million per year, whereas in children is even rarer (0.3 per million per year), being more common in women [1,2]. In contrast, in Southern Brazil, the incidence of ACC in pediatric patients is higher than in other countries and this difference is related to the R337H mutation in the TP53 gene, this genotype being related to aggressive and advanced tumors [1].

The drug 1-(2-chlorophenyl)-1-(4-chlorophenyl)-2,2-dichlorethane (o,p’-DDD), also known as mitotane, was discovered in 1940, as a derivative of the insecticide dichlorodiphenyltrichloroethane (DDT). The adrenolytic effect was first described in dogs, caused by selective necrosis in the fasciculate and reticular zones in the adrenal cortex [3]. The first clinical evidence was published in 1959, showing mitotane effects in ACC [4] and, therefore, mitotane was approved by the FDA for the treatment of ACC. Although the mechanism of action is still not well understood, mitotane remains the main drug currently used for the treatment of postoperative or inoperable ACC, employed as a monotherapy or combined with chemotherapies [5].

Mitotane is administered in doses of 2 to 10 g daily to reach the ideal plasma concentration for the therapeutic effect on ACC [6,7]. In most patients, the stationary phase is reached after three months of daily treatment [8,9]. Although there is controversy over the clinical efficacy of mitotane for increasing patient survival, a meta-analysis shows that mitotane decreases the rate of recurrence and mortality after ACC resection, in patients without distant metastasis [10]. On the other hand, side effects in the gastrointestinal tract reported by patients are diarrhea, nausea, vomiting, anorexia; side effects in the central nervous system are confusion, ataxia, and dizziness [11]. These effects limit the therapeutic use of mitotane, especially in pediatric patients [12]. 

The metabolism of mitotane was previously described via two distinct reactions: α-hydroxylation, which yields the final product o,p’-DDE; and β-hydroxylation, which forms the final product o,p’-DDA [13]. Although the monitoring of o,p’-DDA in the plasma of patients has been demonstrated as a potential prognosis tool [14], it was suggested that the o,p’-DDA is an inactive metabolite [15,16]. In fact, o,p’-DDA is less lipophilic than mitotane or o,p’-DDE, being excreted in the urine [17], which suggests that o,p’-DDA is a final product of the deactivation route. It is speculated that o,p’-DDE may be an active metabolite, since it is not widely found in urine or feces [12] but, despite its low plasma concentration, accumulation of o,p’-DDE has been demonstrated during mitotane treatment [18] and its cytotoxic effect on H295R cells demonstrated [19]. However, no further studies have been developed to confirm this hypothesis. Therefore, considering that o,p’-DDE, along with o,p’-DDA, is an important mitotane metabolite found in the plasma of ACC patients, this study aimed to investigate the action of o,p’-DDE on adrenocortical carcinoma cells.

## 2. Results

### 2.1. Cell Viability of o,p’-DDD and o,p’-DDE in H295R Cell Line

**Figure 1:**
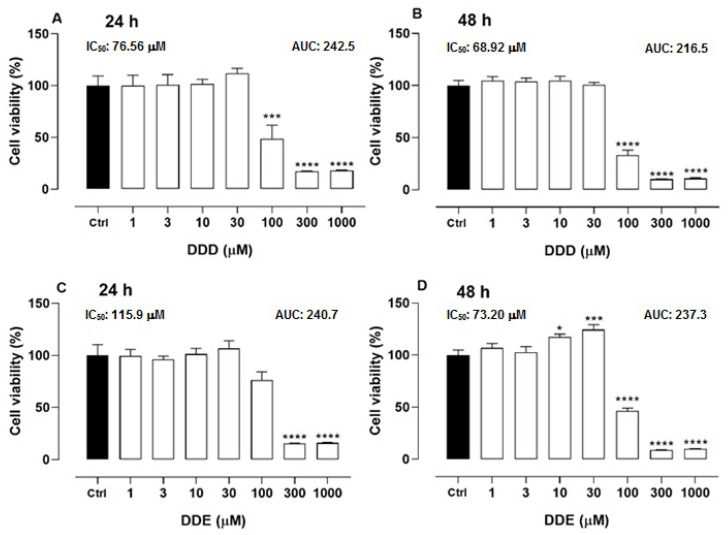
**Effect of o,p’-DDD, and o,p’-DDE on cell viability in H295R cell line.** Cells were incubated with o,p’-DDD or o,p’-DDE at 1, 3, 10, 30, 100, 300, and 1000 µM for 24 (Panels **A**,**C**) and 48 h (Panels **B**,**D**). Ctrl was the vehicle control, containing 1% of ethanol P.A in DMEM F12. Statistical analyses were performed by one-way analysis of variance (ANOVA) followed by Dunnett’s post-test. Data represent the mean ± SEM of three independent experiments (*n* = 6). * *p* < 0.05; *** *p* < 0.0005; **** *p* < 0.0001, compared to the vehicle group (control group).

The H295R cells were incubated with different concentrations of o,p’-DDD. In comparison to untreated cells (control), at concentrations of 100, 300, and 1000 µM, the cell viability was reduced by 51.37, 82.92 and 81.94% in 24 h, and 66.69, 89.99 and 89.21% at 48 h of incubation, respectively (Figure 1A,B). The IC_50_ of o,p’-DDD at 24 h was calculated at 76.56 μM and at 48 h it was 68.92 μM. The cells were also incubated with o,p’-DDE; in 24 h, only concentrations of 300 and 1000 µM were able to reduce the viability, in comparison to the control, by 84.63 and 84%, respectively. In 48 h of incubation, the lower concentrations of o,p’-DDE (10 and 30 µM) seemed to have stimulated cell proliferation, whereas concentrations of 100, 300 e 1000 µM reduced the viability in 53.61, 91.08 and 90.09%, respectively (Figure 1C,D). The calculated IC_50_ of o,p’-DDE was 115.9 μM at 24 h, but in 48 h of incubation, it was obtained at 73.20 μM, being close to the o,p’-DDD.

### 2.2. Effect of o,p’-DDD and o,p’-DDE on Caspase 3/7 Activity in H295R Cell Line

**Figure 2:**
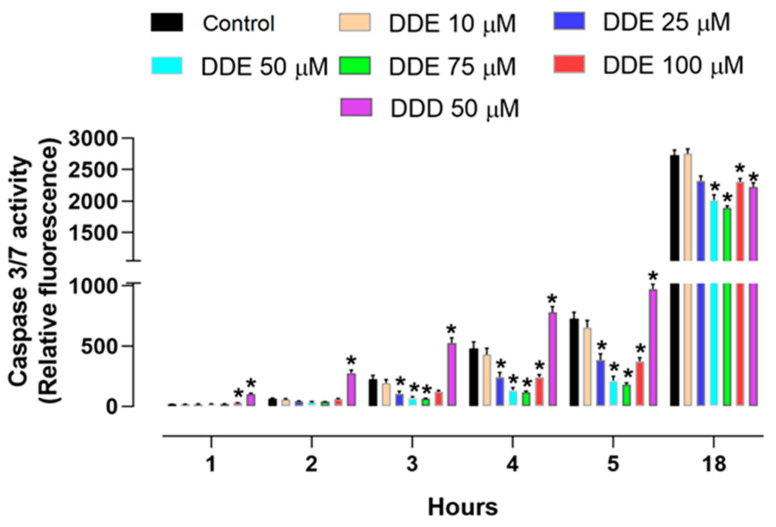
**Caspase 3/7 activity assay.** Cells were incubated with o,p’-DDD at 50 µM and o,p’-DDE at, 10, 25, 50, 75, and 100 µM for 48 h. Control was the vehicle, containing 1% of ethanol P.A in DMEM F12. Statistical analyses were performed by two-way analysis of variance (ANOVA) followed by Dunnett’s multiple comparisons test. Data represent the mean ± SEM of three independent experiments (*n* = 6). * *p* < 0.05 compared to the vehicle group (control group).

H295R cells were incubated with different concentrations of o,p’-DDE or 50 µM of o,p’-DDD for 48 h. After this period, the activity of the caspase 3/7 was monitored at different times (i.e., 1, 2, 3, 4, 5, and 18 h). In comparison to the control, the cells treated with o,p’-DDE had a diminished activity of caspase 3/7, after 3 h, with a reduction of 68.79% and 71.40% with 50 and 75 µM of o,p’-DDE, respectively. After 4 h, the activity decreased by 72.17%, 75.75%, and 46.60%; and in 5 h, to 70.93%, 75.54%, and 48.88%, at the concentrations of 50, 75, and 100 µM, respectively. In comparison to the control, the incubation with o,p’-DDD promoted an increase in the caspase 3/7 activity, by 125.7%, 63.13%, and 33.99% at 3, 4, and 5 h, respectively, contrasting with the effects induced by o,p’-DDE. After 18 h post incubation, the overall activity of caspase 3/7 has increased in comparison to other monitoring times. Nevertheless, by comparison with the control, the o,p’-DDE promote a decrease of 26.03%, 30.7%, and 15.37%, with concentrations of 50, 75, and 100 µM of, respectively. At this time, cells incubated with o,p’-DDD (50 µM), exhibited a modest decrease in the activity of caspase 3/7, being 18.44% less than the control (Figure 2).

### 2.3. Effect of o,p’-DDD and o,p’-DDE on Necroptosis in H295R Cell Line

**Figure 3:**
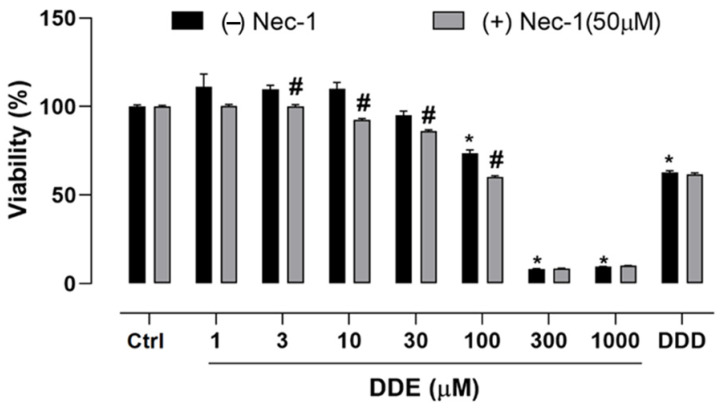
**Necroptosis assay.** Cells were incubated with o,p’-DDD at 50 µM and o,p’-DDE, 1, 10, 30, 100, 300, and 1000 µM for 48 h. Ctrl was the vehicle control, 1% of ethanol P.A in DMEM F12. Statistical analyses were performed by two-way analysis of variance (ANOVA) followed by Tukey’s multiple comparisons test. Data represent the mean ± SEM of three independent experiments (*n* = 6). * *p* < 0.05 compared to the vehicle group (control group). # *p* < 0.05 when comparing (−) Nec-1 and (+) Nec-1.

In the presence (+) or absence (−) of Necrostatin-1 (Nec-1), a RIP1 inhibitor with the capability to prevent the necroptosis process, H295R cells were incubated with different concentrations of o,p’-DDE, or o,p’-DDD at 50 µM for 48 h. The cell viability was monitored by MTT assay, and each group incubated with Nec-1 (+) was compared with those not incubated [(Nec-1 (−)]. A gain in cell viability in the groups Nec-1(+) would indicate the participation of necroptosis in the cell death process, promoted by o,p’-DDE. However, in the groups Nec-1 (+), a slight decrease in cell viability was observed, being more pronounced at 100 µM of o,p’-DDE, which induces a decrease in cell viability of 18.34%, in comparison to group Nec-1 (−). In contrast to the effects of o,p’-DDE, no difference in cell viability was observed in the groups Nec-1 (+) and Nec-1 (−) incubated with o,p’-DDD at 50 µM (Figure 3). This result indicates that the necroptosis process was not involved in the cell death mechanism promoted by o,p’-DDE.

### 2.4. Effect of o,p’-DDD and o,p’-DDE on Necrosis in H295R Cell Line

**Figure 4:**
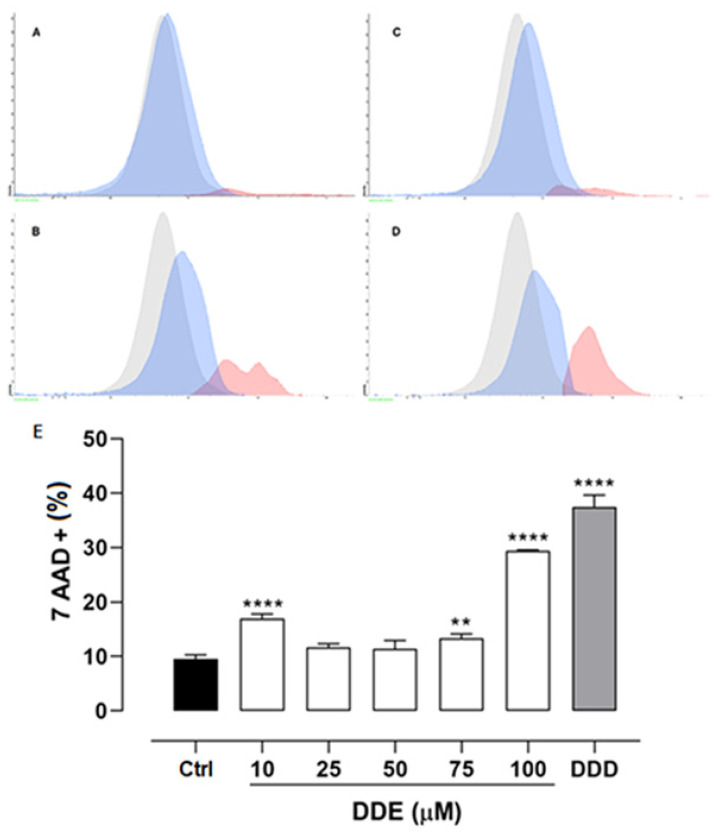
**Necrosis assay.** Cells were incubated with o,p’-DDD at 50 µM and o,p’-DDE 10, 25, 50, 75, and 100 µM for 48 h. Vehicle control (Ctrl) was 1% of ethanol P.A. in DMEM F12. Cytometer histograms of cells treated with Control (**A**), 75 µM of o,p’-DDE (**B**), 100 µM of o,p’-DDE (**C**), and o,p’-DDD at 50 µM (**D**). 7-AAD-positive cells (red), 7-AAD-negative/viable cells (blue), and unstained control cells (gray). Statistical analyses were performed by one-way analysis of variance (ANOVA) followed by Dunnett’s post-test. Data represent the mean ± SEM of three independent experiments (*n* = 3). ** *p* < 0.005; **** *p* < 0.0001, compared to the control group (**E**).

In order to understand the cell death mechanism induced by o,p’-DDE, H295R cells were treated with different concentrations of o,p’-DDE, and 50 µM of o,p’-DDD for comparison. These cell cultures were incubated with 7-aminoactinomycin D (7-AAD), a fluorescent nuclear stain, that is impermeant to the normal plasma membrane of live cells. The plasma membrane of necrotic cells, on the other hand, is permeable to 7-AAD, indicative of an active necrotic process. H295R cells treated with o,p’-DDE at 75 µM increased their fluorescence by 39.37%, but at 100 µM cells exhibited a fluorescence 206.98% higher than that observed in control cells. This confirms that, at least in part, necrosis is a mechanism of cell death induced by o,p’-DDE. Nevertheless, the effect of o,p’-DDD in cell necrosis was higher than that observed with o,p’-DDE, since at 50 µM, the fluorescence of 7-AAD was increased by 291.65%, in comparison with the control group (Figure 4).

### 2.5. Effect of o,p’-DDD and o,p’-DDE on Oxidative Parameters in H295R Cell Line

**Figure 5:**
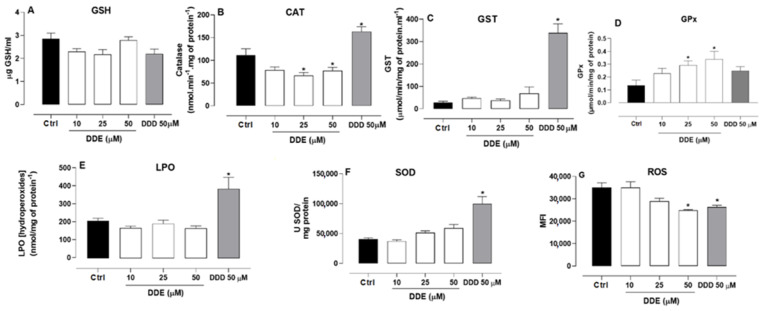
**Oxidative stress.** Cells were incubated with o,p’-DDD at 50 µM and o,p’-DDE at 10, 25, and 50 µM for 48 h and control (1% of ethanol P.A in DMEM F12). Levels of GSH (panel **A**), CAT (panel **B**), GST (panel **C**), GPx (panel **D**), LPO (panel **E**), SOD (panel **F**), and ROS (panel **G**) were measured. Statistical analyses were performed by one-way analysis of variance (ANOVA) followed by Dunnett’s or Holm Sidak’s post-test, when applicable. Data represent the mean ± SEM of three independent experiments (*n* = 6). * *p* < 0.05 compared control group.

The o,p’-DDE at concentrations of 10, 25, and 50 µM, as well as the o,p’-DDD at 50 µM, did not affect the GSH levels in H295R cells, in comparison to untreated cells (Figure 5A). However, in comparison to the control, the o,p’-DDE (25 and 50 µM) induced a decrease in catalase activity, by 40.56% and 30.94%, respectively. By contrast, o,p’-DDD (50 µM) increased catalase activity by 46.05%, in comparison to untreated cells (Figure 5B). The GST was not affected by incubation with o,p’-DDE (Figure 5C), compared to untreated cells; nevertheless, o,p’-DDD induced an increase in GST activity, by more than 10 fold, in comparison to untreated control.

The GPx activity was increased when the cells were treated with 25 and 50 µM of o,p’-DDE, by 115.55% and 151.11%, respectively (Figure 5D), whereas o,p’-DDD did not influence significantly the activity of GPx. No differences were observed in the accumulation of LPO or SOD levels among the untreated cells, and those treated with o,p’-DDE. On the other hand, the cells treated with 50 µM o,p’-DDD, compared to untreated cells, exhibited an increase in these parameters (85.55% for LPO and 146.85% for SOD (Figure 5E and Figure 5F, respectively). The o,p’-DDE, and o,p’-DDD at 50 µM decreased ROS levels by 29.31% and 25.1%, respectively (Figure 5G). 

### 2.6. Effects of o,p’-DDD and o,p’-DDE in Hormone Secretion in H295R Cell Line

**Figure 6:**
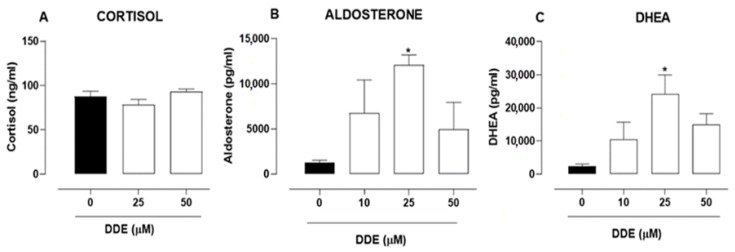
Effects of o,p’-DDE in hormone secretion in H295R cell line. Cells were incubated with o,p’-DDE at 0 (vehicle, 1% of ethanol P.A in DMEM F12), 10, 25, and 50 µM for 48 h. The secretions of cortisol (panel **A**), aldosterone (panel **B**), and DHEA (panel **C**) were measured. Statistical analyses were performed by one-way analysis of variance (ANOVA) followed by Dunnett’s post-test. Data represent the mean ± SEM of three independent experiments (*n* = 3). * *p* < 0.05 compared to the vehicle group (control group).

At the assayed concentrations, the o,p’-DDE did not affect the cortisol secretion in comparison to the control (Figure 6A). However, the o,p’-DDE affected the secretion of aldosterone, increasing it by 858.41% in cells treated with 25 µM (Figure 6B). Similarly, an increase of 465.67% in the DHEA secretion was observed in cells incubated with 25 µM o,p’-DDE, in comparison to untreated cells (Figure 6C). No statistical significance was obtained with other concentrations of o,p’-DDE.

### 2.7. Molecular Docking of o,p’-DDE with CYP11A1, CYP11B1, CYP21A2, CYP19A1 and CYP17A1

**Figure 7:**
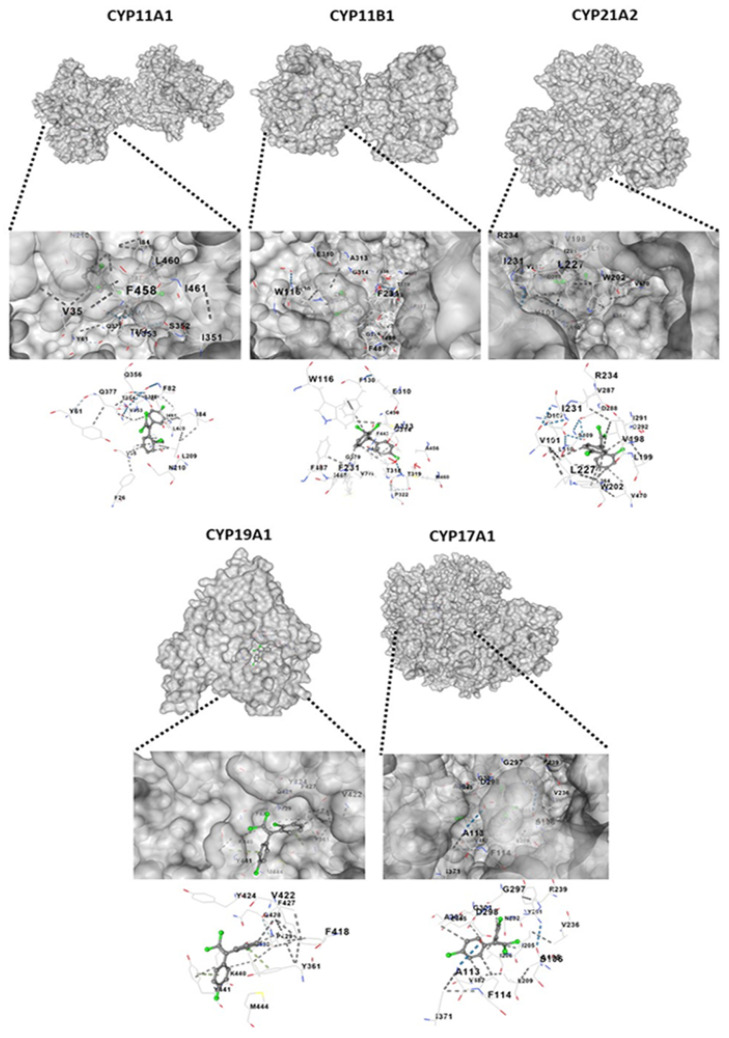
Molecular docking of o,p’-DDE with CYP11A1, CYP11B1, CYP21A2, CYP19A1 and CYP17A1.

The Vina scores, cavity sizes, docking centers, and sizes of the predicted cavities from the docking simulation of o,p’-DDE for different CYPs are shown in Table 1. A negative value of the Vina score represents a more stable binding of the compound to the target. High values for cavity size indicate that the cavity size is close to or larger than the compound, resulting in the accuracy of docking [20,21]. All docking sketch maps of the target protein with o,p’-DDE are shown in Figure 7.

### 2.8. Effects of o,p’-DDD and o,p’-DDE in CYP’s Expression

**Figure 8:**
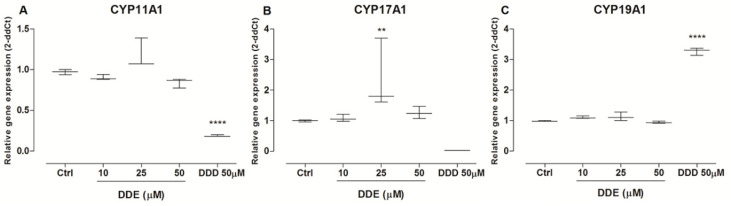
**Gene expression of o,p’-DDD, and o,p’-DDE in H295R cell line**. Cells were incubated with o,p’-DDD at 50 µM and o,p’-DDE at, 10, 25, and 50 µM for 48 h. The vehicle control (Ctrl) was 1% of ethanol P.A in DMEM F12. Expression of *CYP11A1* (panel **A**), *CYP17A1* (panel **B**), and *CYP19A1* (panel **C**) was determined. Statistical analyses were performed by Kruskal–Wallis test followed by Dunn’s Multiple Comparison post-test. Data represent the mean ± SEM of three experiments (*n* = 3). ** *p* < 0.005; **** *p* < 0.0001, compared to vehicle group (control group).

Mitotane at a concentration of 50 µM decreased the expression of *CYP11A1* and *CYP17A1*, by 82% and 98%, respectively, in comparison to untreated cells. On the other hand, by comparison to the control cell, the mitotane increased the gene expression *CYP19A1* by 229% (Figure 8A,C). For cells treated with o,p’-DDE, the expression of *CYP11A1* and *CYP19A1* had no differences compared to the control group, but there was an increase of 70% in the expression of *CYP17A1* at the intermediate concentration of 25 µM (Figure 8B). 

## 3. Discussion

The compounds o,p’-DDE, and o,p’-DDA are the major products of mitotane metabolism, raising doubts if metabolic activation is important for the therapeutic effect [15]. Despite its lower plasma concentration, it has been discussed whether o,p’-DDE could be an active metabolite since it displays cytotoxic effects on the adrenocortical cell line (H295R) [12]. Higher concentrations of o,p’-DDE are found associated with chylomicrons, and this could explain its plasma-free lower concentration [17]. Moreover, o,p’-DDE is not found in feces or urine, indicating it has a cumulative behavior in patients, over the therapy [18]. As a prognosis tool, a previous study reported the medians of o,p’-DDE levels in plasma, finding 2.87 µM for responders, 2.03 µM for patients with stable disease, and 1.56 µM for non-responders [14]. Herein, we observed that o,p’-DDE displayed similar cytotoxicity as mitotane, in H295R cells, after 48 h of incubation, with close IC_50_ values. This agrees with a previous study, where o,p’-DDE also had cytotoxic effects in the H295R cell line, after 72 h of incubation [18]. Furthermore a similar compound, MeSO_2_-DDE, assayed at 5, 10, and 15 µM, also reduced significantly the viability of H295R cells after 72 h of incubation [22]. Despite this cytotoxic effect on the adrenal cell line, the mechanism of action displayed by the o,p’-DDE was not determined. Therefore, we investigated some cell death pathways, such as apoptosis, necroptosis, ferroptosis, and necrosis, to explain the effects of o,p’-DDE.

It has been found that mitotane induces apoptosis in H295R cells, increasing caspase 3/7 activity [23,24,25,26]. Accordingly, we observed an increase in the caspase 3/7 activity in H295R cells incubated with mitotane at 50 µM (in comparison to untreated control), confirming apoptosis as an important pathway of cell death. On the other hand, in comparison to untreated cells, o,p’-DDE significantly inhibited the caspase 3/7 activity at almost all concentrations assayed (Figure 2), indicating a different cell death pathway, other than apoptosis. Necroptosis is a highly regulated type of necrosis [27], that occurs through the activation of the receptor-interacting protein (RIP), RIPK1 and RIPK3, and mixed lineage kinase domain-like pseudokinase (MLKL) [28,29]. However, the o,p’-DDE was able to decrease the H295R cell viability, even in the presence of Nec-1, an inhibitor of the necroptosis pathway, as also observed after incubation with mitotane. Therefore, no involvement of necroptosis in the cell death mechanism of mitotane or o,p’-DDE was observed. Instead, the combination of o,p’-DDE, and Nec-1, induced higher cytotoxicity in H295R cells, than o,p’-DDE.

The treatment with o,p’-DDE increased the GPx activity (Figure 5D), indicating an inhibition of the ferroptosis pathway. Furthermore, the evolution of ferroptosis is accompanied by increased lipid peroxidation (LPO) and ROS production [30,31], which did not occur in the cells treated with o,p’-DDE (Figure 5E,G). Mitotane did not induce ferroptosis [30,32], which might explain why GPx levels did not decrease after exposure to mitotane (Figure 5D). A depletion in GSH impairs the activity of GPX4, resulting in an accumulation of peroxidized lipids which are involved in the ferroptosis mechanism [33]. Herein, both mitotane and o,p’-DDE had no effects in GSH levels (Figure 5A). However, mitotane at 50 µM increased the lipid peroxidation in H295R cells, in comparison to untreated cells, which was expected since the drug promotes strong induction of lipid peroxidation in ACC cells [30]. By contrast, the treatment of H295R cells with o,p’-DDE did not lead to an LPO accumulation, also indicating that ferroptosis is not involved in the cell death mechanism. Instead, as in mitotane incubation, a significant increase of 7-AAD staining was observed in cells treated with 100 μM o,p’-DDE, in comparison to the untreated control, revealing that cells treated at high concentrations of o,p’-DDE undergo a necrotic process.

Superoxide dismutase (SOD) and Catalase (CAT) are important enzyme classes in cell oxidative stress. Whereas SOD promotes superoxide dismutation, the CAT reacts with H_2_O_2_ to form water and oxygen [34,35,36]. As previously observed [16], in our assays, mitotane stimulated the SOD expression in the H295R cells, but the cells treated with o,p’-DDE displayed only a discrete increase (without statistical significance) in SOD activity. Interestingly, another mitotane metabolite, o,p’-DDA, also did not promote a stimulatory effect in the SOD activity in H295R cells [16]. Mitotane also stimulated the CAT expression, which can protect cells from oxidative damage; notwithstanding, the o,p’-DDE slightly decreased the CAT activity in comparison to the control (Figure 8B). The increase in CAT and SOD activity exerted by mitotane treatment may explain the diminished levels of ROS observed in H295R cells, in comparison to the untreated cells (control). On the other hand, despite o,p’-DDE depleting CAT expression, a decreased level of ROS was observed in the cells treated with o,p’-DDE in comparison to the control. Therefore, o,p’-DDE was unable to stimulate SOD, as mitotane did, and it promoted a depletion in the CAT expression. Nevertheless, at 50 μM, both mitotane and o,p’-DDE were able to deplete the ROS levels equivalently, suggesting there is no involvement of oxidative stress in the mechanism of cell death of o,p’-DDE.

GST has a pivotal role in cell detoxification against xenobiotics. The expression of GST in H295R cells incubated with mitotane 50 μM was augmented about 12-fold, in comparison to the untreated cells (control), indicating that mitotane can be conjugated with GSH for being eliminated. However, the o,p’-DDE did not stimulate the expression of GST as observed with mitotane, but a slight augmentmentation was observed with 50 μM of o,p’-DDE; in comparison to untreated cells, this was not statistically significant. Therefore, in contrast to mitotane, the o,p’-DDE seems not to be eliminated via the GSH-GST system.

Metabolic activation of mitotane may be dependent on CYP11B1, or others CYPs, and the ability of the tumor to metabolize mitotane may predict the response to treatment [15]. In comparison to untreated cells, mitotane decreased the expression of CYP11A1 and CYP17A1 (Figure 8A,B), as previously observed [24,37]. Nevertheless, it increased the expression of CYP19A1 which, to our knowledge, was not previously described. Thus, we investigated the potential of o,p’-DDE to induce the activity of CYPs involved in the production of aldosterone and DHEA, which could explain the increased levels of these hormones, in comparison to untreated cells (Figure 6B,C). Indeed, our in silico analysis, following the Vina score and cavity size, showed a strong interaction between o,p’-DDE, and CYP11A1, CYP11B1, CYP21A2, CYP19A1 and, mainly, with CYP17A1, which had the highest value of cavity size. Furthermore, in comparison to the control, the o,p’-DDE, at 25 µM, showed the higher stimulation of CYP17A1 expression (Figure 8B), confirming their close relationship, as observed in our in silico assays. CYP17A1 converts the 17-hydroxypregnenolone to DHEA, explaining the increased concentrations of DHEA in H295R cells treated with o,p’-DDE (Figure 6C), compared to untreated control.

A previous investigation showed that MeSO_2_-DDE, at the lower concentration, stimulated steroid production and CYP11B1 expression, but had the opposite effect when assayed at higher concentrations [22], suggesting a biphasic response which is reported with a wide variety of compounds [12,37,38]. This behavior is called hormesis, a dose-response relationship characterized by low-dose stimulation and high-dose inhibition [39]. In H295R cells, 1.25 μM of MeSO_2_-DDE increased the levels of CYP11B1, CYP11B2, and StAR, whereas concentrations of 5 and 10 μM did not [39]. A similar biphasic response was observed in steroid secretion; the lower concentrations of MeSO_2_-DDE (at 1.25 µM) increased cortisol and aldosterone levels, whereas at 10 µM the steroid hormones secretion was reduced [39,40]. Herein, we observed that o,p’-DDE stimulated the expression of CYP17A1 and increased aldosterone and DHEA secretion, at the intermediate concentration assayed (25 µM) whereas the levels of cortisol remained unchanged. The stimulation promoted by o,p’-DDE occurred in the intermediate concentration investigated, whereas the lower and higher concentrations did not affect the hormone secretion nor the CYPs expression, being consistent with a biphasic behavior, as described for MeSO_2_-DDE.

## 4. Materials and Methods

### 4.1. Cell Culture

NCI-H295R cells (ATCC, Manassas, VA, USA) were maintained in Dulbecco′s Modified Eagle′s Medium/Ham′s Nutrient Mixture F12 (DMEM F12) (Sigma-Aldrich, St. Louis, MO, USA), 10% of fetal bovine serum (FBS), and 1% Penicillin/Streptomycin (GIBCO, Carlsbad, CA, USA) at 37 °C and 5% CO_2_ [41]. For the experiments with H295R, the cells were used between the 5 and 10 passages as recommended by ATCC (CLR-2128, 2007), to ensure the secretion of steroid hormones.

### 4.2. Cell Viability

The H295R cells were seeded at a concentration of 1 × 10^4^ in a 96-well plate and incubated for 24 h at 37 °C and 5% CO_2_. After that, the cells were exposed to o,p’-DDD, and o,p’-DDE at concentrations of 0, 1, 3, 10, 30, 100, 300, and 1000 µM, diluted in 1% of ethanol P.A. (Sigma-Aldrich, St. Louis, MO, USA) and DMEM F12. At 24 and 48 h post-incubation, the culture supernatant was removed and 100 µL of MTT (Invitrogen, Carlsbad, CA, USA) solution (0.5 mg/mL) in DMEM F12 was added to each well. After 3 h of incubation at 37 °C the supernatant was removed and 100 µL of dimethyl sulfoxide (DMSO) was added and the plate was kept in agitation for 5 min, protected from light [42]. Finally, the absorbance at 595 nm was measured by a microplate reader EPOCH BIOTEK^®^ (BIOTEK, Winooski, VT, USA). The IC_50_ values were determined by the Software GraphPad Prism, three or five concentrations of o,p’-DDE and one concentration of o,p’-DDD were chosen for the next experiments.

### 4.3. Caspase 3/7 Assessment

The cells were seeded at a concentration of 5 × 10^4^ in a black 96-well plate. After 24 h of incubation, the supernatant was removed and 0, 10, 25, 50, 75, and 100 µM of o,p’-DDE, and 50 µM of o,p’-DDD were added, in sextuplicate. After 48 h of incubation at 37 °C and 5% CO_2_, 100 µL of the caspase 3/7 kit (Apo-ONE^®^) was added to the cells, following the manufacturer’s instructions (Promega, Madison, WI, USA). Fluorescence was monitored in a Synergy LX Multimode Reader (BIOTEK, Winooski, VT, USA) at 499/521 nm. The results were expressed in relative fluorescence. 

### 4.4. Necroptosis Assessment 

The cells were seeded at a concentration of 5 × 10^4^ in two 96-well plates. After 24 h, the supernatant was removed and cells were treated with o,p’-DDE at 0,1, 3, 10, 30, 100, 300, and 1000 µM and 50 µM of o,p’-DDD, and incubated for 48 h. Then, 100 µL of MTT solution (0.5 mg/mL) in DMEM F12 was added to each well. In one plate 50 mM of Necrostatin-1 (Nec-1) (Sigma-Aldrich, St. Louis, MO, USA), an inhibitor of receptor-associated kinase 1 (RIPK1), was added. The plates were incubated for 3 h at 37 °C and the same cell viability protocol was performed, as described previously in item 4.3. The plate was read at 595 nm by a microplate reader EPOCH BIOTEK^®^ (BIOTEK, Winooski, VT, USA). 

### 4.5. Necrosis Assessment

The cells were seeded at a concentration of 1 × 10^6^ in a 6-well plate. After 24 h, the supernatant was removed and the cells were treated with o,p’-DDD at 50 µM and o,p’-DDE at 0, 10, 25, 50, 75, and 100 µM, and incubated for 48 h. After incubation, the supernatant was collected and cells were washed with 1 mL of PBS and trypsinized. The cells were mixed with the supernatant to maintain dead cells. The cells were centrifuged at 1400 rpm for 10 min and resuspended in PBS. The cells were transferred in cytometer tubes and 400 µL of PBS and 5 µL of 7-AAD (BD Pharmingen, Franklin Lakes, NJ, USA) were added. The samples were incubated for 15 min at room temperature and analyzed in a flow cytometer FACSCanto II (Becton Dickinson, Franklin Lakes, NJ, USA).

### 4.6. Oxidative Parameters 

H295R cells were seeded at a concentration of 1 × 10^6^ in a 6-well plate until reaching the confluence of 80–90% [43]. After that, the cells were incubated with 0, 10, 25, and 50 µM of o,p’-DDE, and 50 µM of o,p’-DDD for 48 h [44]. After incubation, cells were washed with cold PBS, trypsinized, and centrifuged for 5 min at 1400 rpm and 4 °C. The supernatant was removed and the cells were resuspended in 1 mL of PBS. The cells were centrifuged at 1400 rpm for 5 min at 4 °C and used for reduced glutathione assay (GSH) and lipoperoxidation assay (LPO). An aliquot of the supernatant was collected and stored at −80 °C for further experiments of SOD, CAT, protein, GST, and GPx, described below. 

#### 4.6.1. Reduced Glutathione (GSH)

To measure the content of reduced Glutathione (GSH) in the H295R cell line, 80 µL of ATC 12.5% was added to 100 µL of the homogenates and the mixture was homogenized. The samples were centrifuged at 6000 rpm for 15 min at 4 °C. After that, 50 µL of the supernatant was added in triplicate in a 96-well plate and 250 µL of TRIS-HCl (0.4 M) (Sigma-Aldrich, St. Louis, MO, USA) was added in each well. Then, 5 µL of DTNB was added to each well and the plate was incubated for 5 min, protected from light. The samples were read at 415 nm by a microplate reader EPOCH BIOTEK^®^ (BIOTEK, Winooski, VT, USA). The same procedure was performed for the standard curve (1.25, 2.5, 5, 7.5, and 10 µg/mL) [45].

#### 4.6.2. Catalase (CAT)

Catalase activity was measured by adding 5 µL of the supernatant in a 96-well plate in triplicate and 295 µL of hydrogen peroxide solution (Sigma-Aldrich, St. Louis, MO, USA) was added in each well, horizontally and in a row at a time. The kinetics of the reaction was read at 240 nm by a microplate reader EPOCH BIOTEK^®^ (BIOTEK, Winooski, VT, USA) and the results were expressed in activity catalase specific (nmol·min^−1^·mg proteins^−1^) [46].

#### 4.6.3. Glutathione S-Transferase (GST)

The Glutathione S-transferase (GST) activity was determined by adding 100 µL of the supernatant in a 96-well plate in triplicate with 200 µL of reaction solution (CDNB and GSH). The kinetics of the reaction was read at 340 nm by a microplate reader EPOCH BIOTEK^®^ (BIOTEK, Winooski, VT, USA) and the results were expressed in U of activity/protein mg [47].

#### 4.6.4. Lipoperoxidation Assay (LPO)

For the analysis of lipoperoxidation (LPO), the homogenates were resuspended in methanol (Sigma-Aldrich, St. Louis, MO, USA) (1:1) and the samples were centrifuged at 8000× *g* (RCF) for 5 min at 4 °C. Then, 100 µL of the samples were added to 900 µL of FOX-2 solution and the solution was homogenized and incubated for 30 min, protected from light. After incubation, 300 µL of the samples were added to a 96-well plate in triplicate and read at 560 nm by a microplate reader EPOCH BIOTEK^®^ (BIOTEK, Winooski, VT, USA) [48].

#### 4.6.5. Superoxide Dismutase (SOD)

To measure the superoxide dismutase (SOD) activity, 60 µL of the supernatant was added in microtubes with, 1.3275 µL of Tris-EDTA Buffer (Sigma-Aldrich, St. Louis, MO, USA), and the samples were homogenized. After that, 75 µL of pyrogallic acid (Sigma-Aldrich, St. Louis, MO, USA) was added and after homogenization the samples were incubated for 30 min, protected from light. To stop the reaction, 37.5 µL of HCl 1N was added to the solution. 300 µL of the samples were transferred, in triplicate, to a 96-well plate and read at 440 nm by a microplate reader EPOCH BIOTEK^®^ (BIOTEK, Winooski, VT, USA) [49].

#### 4.6.6. Reactive Oxygen Species (ROS)

To measure the total reactive oxygen species (ROS), the cells were seeded at a concentration of 1 × 10^6^ in a 6-well plate and incubated for 24 h. The cells were treated with 10, 25, and 50 µM of o,p’-DDE, and 50 µM of o,p’-DDD, for 48 h. After incubation, the supernatant was collected and cells were washed with 1 mL of PBS and trypsinized with trypsin-EDTA solution (GIBCO, Carlsbad, CA, USA). The cells were mixed in the supernatant to maintain dead cells. The cells were centrifuged at 1400 rpm for 10 min and resuspended in PBS. The cells were transferred into cytometer tubes and 500 µL of PBS with DCFH-DA (Sigma-Aldrich, St. Louis, MO, USA) was added, at a concentration of 35 µM, and the tubes were incubated for 30 min at 37 °C. The samples were analyzed in a flow cytometer FACSCanto II (Becton Dickinson, Franklin Lakes, NJ, USA) [50].

#### 4.6.7. Glutathione Peroxidases (GPx)

For Glutathione peroxidases (GPx) analysis, 10 µL of the samples were added in triplicate in a 96-well plate with 130 µL of a solution with NaN_3_, NADPH, GSH, and GR (all from Sigma-Aldrich, St. Louis, MO, USA) and the plate was incubated for 2 min. After incubation, 60 µL of hydrogen peroxide solution was added and the kinetics of the reaction was read at 340 nm by a microplate reader EPOCH BIOTEK^®^ (BIOTEK, Winooski, USA). The results were expressed in µmol·min^−1^ mg protein^−1^ [51].

#### 4.6.8. Protein Quantification

To measure the protein content, 10 µL of the supernatant was added in triplicate in a 96-well plate with 250 µL of Bradford solution (Sigma-Aldrich, St. Louis, MO, USA) and the plate was read at 595 nm by a microplate reader EPOCH BIOTEK^®^ (BIOTEK, Winooski, VT, USA). The result was normalized by the protein standard curve (BSA at 125, 250, 500, and 1000 µg mL^−1^). The same procedure was performed for the standard curve [52].

### 4.7. Dosage of DHEA, Cortisol and Aldosterone

The cells were seeded at a concentration of 3 × 10^4^ in a 24-well plate. After 24 h, the supernatant was removed and the cells were incubated with 10, 25, and 50 µM of o,p’-DDE, for 48 h. After that, the supernatant was collected and the samples were stored at −80 ºC. Cortisol, aldosterone, and DHEA levels were determined by ELISA kits following producer instructions (Invitrogen, Carlsbad, CA, USA).

### 4.8. Molecular Docking

The molecular docking simulation was conducted using a method based on cavity detection by CB-Dock (http://cao.labshare.cn/cb-dock/; accessed on 15 October 2022) [20]. The crystal structures of the CYP11A1, CYP11B1, CYP21A2, CYP19A1, and CYP17A1 were downloaded from the protein data bank (http://www.rcsb.org; accessed on 15 October 2022). The 3D structure of o,p’-DDE was downloaded from the PubChem compound database (https://pubchem.ncbi.nlm.nih.gov/; accessed on 15 October 2022) and converted to “.mol” extension. Then, the crystal structures of proteins and the 3D structure of o,p’-DDE were inputted to CB-Dock and binding activities were analyzed according to vina score, cavity size, docking center, and size [20].

### 4.9. CYP’s Expression

The cells were seeded at a concentration of 1 × 10^6^ in a 6-well plate. After 24 h, the supernatant was removed and the cells were incubated with 10, 25, and 50 µM of o,p’-DDE, and 50 µM of o,p’-DDD, in triplicate, for 48 h. Following incubation, total RNA was extracted from the cells using the GenElute™ Total RNA Purification Kit, following the manufacturer’s instructions (Sigma-Aldrich, St. Louis, MO, USA). 1 µg of the extracted RNA was reverse-transcribed using High-Capacity cDNA Reverse Transcription Kit (Invitrogen, Carlsbad, CA, USA). The cDNAs were amplified by quantitative PCR using the primers for *CYP11A1* (Hs00167984), *CYP17A1* (Hs01124136), and *CYP19A1* (Hs00903411) (Thermo Fisher Scientific, Waltham, MA, USA). Gene expression levels were calculated relative to their respective control GAPDH and expressed as 2-ddCt.

### 4.10. Statistical Analysis

All data were expressed as means ± standard error of the mean (S.E.M.). Results were subjected to the Kolmogorov–Smirnov normality test and by one or two-way analysis of variance (ANOVA), followed by post hoc of Dunnett’s, Tukey’s or Holm–Sidak’s multiple comparisons test, when applicable. For results that failed on the normality test, the Kruskal–Wallis test with Dunn’s Multiple Comparison Test were performed. The results were analyzed using GraphPad Prism (v. 5.0, San Diego, CA, USA). The level of significance was set at 95% (*p* < 0.05).

## 5. Conclusions

Despite the speculation over the o,p’-DDE effects on patients during mitotane therapy, few studies were performed to evaluate the action of o,p’-DDE in the adrenocortical cells. Therefore, in this study, we investigated the cell pathway using the H295R cell line and found that necrosis was the main mechanism of cell death promoted by o,p’-DDE. We also reported, for the first time, a biphasic-like behavior of H295R cells against o,p’-DDE, as observed in the hormone secretion effects and stimulation of CYP17A1, which explained the DHEA secretion profile after incubation with different concentrations of o,p’-DDE. Therefore, due to these important findings, and its cumulative behavior, the levels of o,p’-DDE in plasma patients should also be monitored during the mitotane therapy to ensure efficacy and safety to patients.

## Figures and Tables

**Table 1:** Vina scores and cavity information of the docking simulation pose for CYPs and o,p’-DDE.

Receptor	PDB ID	Vina Score	Cavity Size	Center			Size		
				x	y	z	x	y	z
CYP11A1	3N9Y	−7.9	3903	14	−5	17	19	31	19
CYP11B1	6M7X	−7.5	2562	51	−47	−8	25	27	34
CYP21A2	4Y8W	−8	2231	−13	12	28	25	19	19
CYP19A1	5JL6	−7	2120	84	51	49	19	19	30
CYP17A1	3RUK	−7.5	19,788	26	16	43	35	35	35

## Data Availability

Data is contained within the article.
